# Long-term dynamics of trematode infections in common birds that use farmlands as their feeding habitats

**DOI:** 10.1186/s13071-021-04876-2

**Published:** 2021-08-06

**Authors:** Jiljí Sitko, Petr Heneberg

**Affiliations:** 1 Comenius Museum, Moravian Ornithological Station, Přerov, Czech Republic; 2 Charles University, Third Faculty of Medicine, Prague, Czech Republic

**Keywords:** Agricultural landscapes, Common farmland birds, Biodiversity decline, Helminths, Population dynamics, Trematoda

## Abstract

**Background:**

The biodiversity of farmland habitats is witnessing unprecedented change, mostly in declines and simplification of assemblages that were established during centuries of the use of traditional agricultural techniques. In Central Europe, conspicuous changes are evident in populations of common farmland birds, in strong contrast to forest birds in the same region. However, there is a lack of information on longitudinal changes in trematodes that are associated with common farmland birds, despite the fact that diversity of trematodes is directly linked to the preservation of long-established food webs and habitat use adaptations of their hosts.

**Methods:**

We analyzed the population trends of trematodes for the period 1963–2020 in six bird species that use Central European farmlands as their predominant feeding habitats. Namely, we examined *Falco tinnunculus*, *Vanellus vanellus*, winter populations of *Buteo buteo*, *Ciconia ciconia*, extravilan population of *Pica pica*, and *Asio otus*, all originating from the Czech Republic.

**Results:**

We observed dramatic population losses of all trematode species in *C. ciconia* and *V. vanellus*; the changes were less prominent in the other examined hosts. Importantly, the declines in prevalence and intensity of infection affected all previously dominant species. These included *Tylodelphys excavata* and *Chaunocephalus ferox* in *C. ciconia*, *Lyperosomum petiolatum* in *P. pica*, *Strigea strigis* in *A. otus*, *Neodiplostomum attenuatum* and *Strigea falconis* in *B. buteo* (*χ*^2^ test *P* < 0.001 each), and *Echinoparyphium agnatum* and *Uvitellina adelpha* in *V. vanellus* (completely absent in 2011–2000). In contrast, the frequency and spectrum of isolated records of trematode species did not change to any large extent except those in *V. vanellus*.

**Conclusions:**

The analysis of six unrelated common bird species that use farmlands as their feeding habitats revealed a previously unreported collapse of previously dominant trematode species. The previously dominant trematode species declined in terms of both prevalence and intensity of infection. The causes of the observed declines are unclear; of note is, however, that some of the broadly used agrochemicals, such as azole fungicides, are well known for their antihelminthic activity. Further research is needed to provide direct evidence for effects of field-realistic concentrations of azole fungicides on the survival and fitness of trematodes.

**Graphical abstract:**

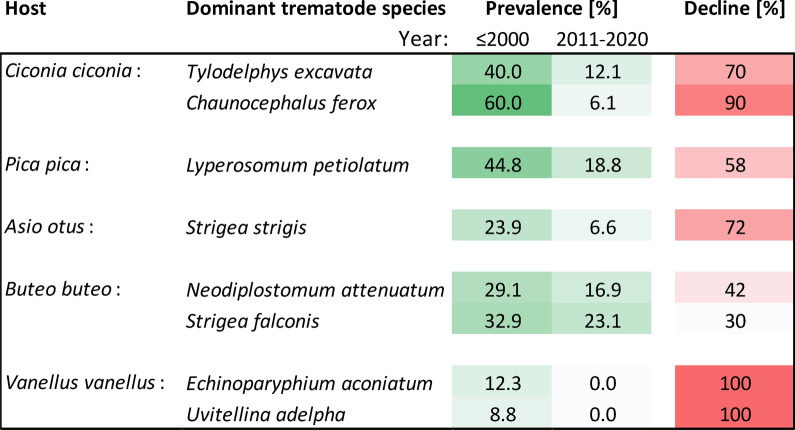

**Supplementary Information:**

The online version contains supplementary material available at 10.1186/s13071-021-04876-2.

## Background

Metazoan parasites are considered one of the most diverse, threatened, and under-protected animals on Earth [1]. The roles of parasites in the ecosystem remain understudied and underappreciated [2]. Moreover, only a very small number of parasite species have well-documented distributions or population sizes [1]. However, the identification of species that are at the greatest risk of extinction requires the availability of robust data, particularly the evidence of changes in diversity and/or distribution in time [3]. The rare species are not necessarily equal to threatened species. Therefore, evaluation of the conservation status of threatened taxa requires knowledge not only of the rarity of the respective species but also of the population trends or habitat deterioration. The evidence for population size reduction over a period of ≥ 10 years or ≥ 3 generations represents one of the three major criteria for the inclusion of the respective species as threatened (category Vulnerable or higher) in the International Union for Conservation of Nature (IUCN) Red List (https://www.iucnredlist.org/resources/summary-sheet). Concerning the trematode bird host species in the study area, in central Europe, the most conspicuous population changes are experienced by common farmland birds, whereas the populations of forest birds remain relatively stable or increasing [4, 5]. Longitudinal data on trematode assemblages are scarce, and the trematodes of farmland birds have never been subjected to a longitudinal study. Previous studies have addressed trematode diversity in aquatic birds that serve as definitive hosts of trematodes [4, 5] and in snail intermediate hosts [6–10]. The situation may differ regionally and the declines may be habitat-specific. The studies that were performed in aquatic habitats revealed that trematodes face an unprecedented decline in terms of both their abundance and species richness in Western countries, but not so in less exploited regions, such as Siberia [6–12]. However, some host species living in the industrialized landscapes, such as *Turdus philomelos*, displayed increases in the diversity of helminths during recent decades [13].

The bird species that use farmlands as their predominant feeding areas differ in their spatiotemporal distribution. These species include (1) common farmland birds, such as *Falco tinnunculus* and *Vanellus vanellus*, (2) species which utilize the farmlands nearly exclusively during the winter months, such as *Buteo buteo*, and (3) species that use farmlands as their main feeding habitats, such as *Ciconia ciconia*, extravilan (living outside the city/village limits) populations of *Pica pica*, and *Asio otus*. These species overlap only partially in their food preferences, but all their food is, in general, affected by the broad-spectrum pesticides applied to the agricultural crops [14–16], which therefore may affect the complex life cycles of the trematodes. During the period 1982–2005, the Czech populations of these six species experienced the following population trends: *Ciconia ciconia* stable, +1.9%; *P. pica* stable, +1.7%; *A. otus* declining, −4.0%; *B. buteo* stable, +1.4%; *F. tinnunculus* stable, 0.0% (but declined by 35% in 1980–2003 in Europe); and *V. vanellus* declining, −9.9% (also declined by 51% in 1980–2003 in Europe) [17, 18]. The studies from Russia and Central Asian countries from the 1950s, studies from Central Europe from the 1960s, and studies from other parts of Europe from the recent decades have provided trematode prevalence data from large data sets of these six bird host species (Additional file 1: Tables S1–S6). However, differences in the geographic origin of the data make it difficult to estimate prevalence trends in any of the trematodes. See, for example, the below-listed pairs of studies from different geographic regions, which reported conflicting data concerning the prevalence and species richness of trematodes in *P. pica* [19, 20] (Additional file 1: Table S2), *B. buteo* [21, 22] (Additional file 1: Table S4), or *V. vanellus* [23, 24] (Additional file 1: Table S6). No longitudinal studies of trematodes have been reported in any of the six species.

In the present study, we address long-term changes in component communities of trematodes in six bird species which feed over the nesting season (five species) or in winter only (one species) in open farmland habitats. We collected the source data across a time span of over half a century. The adults might harbor helminths that were acquired during migration and at their wintering grounds, and the nestlings are often fed different types of food compared to the full-grown birds. Therefore, we analyzed the component communities of first-year birds and those of adult females or males separately.

## Methods

For the period 1963–2020, we examined 147 individuals of white stork *C. ciconia*, 169 individuals of Eurasian magpie *P. pica*, 242 individuals of long-eared owl *A. otus*, 259 individuals of common buzzard *B. buteo*, 1092 individuals of common kestrel *F. tinnunculus*, and 78 individuals of northern lapwing *V. vanellus* for the presence of trematodes. The examined birds were stratified according to their age to first-year birds (1Y; born in the calendar year that they were examined) and adults (birds in their second or later calendar year of life) [25, 26]. The adults were further stratified according to their sex. To enable the adult and 1Y *P. pica* to be merged in our analyses, we identified the sex of both adult and 1Y individuals of this species. In another species with low numbers of 1Y birds, *V. vanellus*, we did not keep records of the sex of the 1Y birds that we examined in the 1960s and 1970s. To analyze the changes in analyzed helminth assemblages over time, we split the obtained bird hosts into groups according to the time when they were obtained. As the availability of the respective species was not equal across the study period, the chosen time intervals were set individually for each of the six examined bird host species (Table 1). Concerning *B. buteo*, we examined only adult birds obtained in winter months from areas without larger forests; in winter months, *B. buteo* characteristically occurs in open habitats.

**Table 1:** Numbers of host individuals that were examined in the present study

Species	Number of individuals		
Study period	1Y	Adult F	Adult M
*Ciconia ciconia*			
1963–2000	18	9	8
2001–2010	30	4	12
2011–2020	45	12	9
*Pica pica*^a^			
1991–2000	7	20	31
2001–2010	4	6	16
2011–2020	1	33	51
*Asio otus*			
1963–1990	5	29	11
1991–2000	14	22	11
2001–2010	10	29	35
2011–2020	20	29	27
*Buteo buteo*			
1981–1990		21	18
1991–2000		102	17
2001–2010		19	17
2011–2015		14	10
2019–2020		29	12
*Falco tinnunculus*			
1991–1995	16	16	18
1996–2000	26	76	59
2001–2005	25	47	43
2006–2010	75	123	53
2011–2015	53	125	96
2016–2020	59	112	70
*Vanellus vanellus*			
1963–1980	3	13	29
1981–2000	4	1	7
2001–2020	4	4	13

^a^Because of the low numbers of 1Y *P. pica*, the sex of the 1Y *P. pica* was identified and these individuals were analyzed together with the adults. The respective individuals can be tracked in Additional file 1

All the examined birds originated from the eastern and southern Czech Republic (48.7° N–49.80° N, 13.3° E–18° 30′ E). We obtained the dead birds before they were prepared for the Comenius Museum collection (Přerov, Czech Republic). The birds consisted primarily of wounded, hunted, or injured individuals, most of which were euthanized in rescue stations due to untreatable wounds. Concerning birds provided by the rescue stations, these included only individuals that were not treated with antihelminthic agents prior to being euthanized. As an exception, the examined *P. pica* were provided by local hunters; *P. pica* is listed as game according to the Decree of the Ministry of Agriculture No. 245/2002 Coll. and can be legally hunted without restriction in the period from July 1st to February 28th. Our long-term research was authorized by governmental and local authorities; our most recent permit was issued by the Ministry of the Environment of the Czech Republic on August 3, 2009 under No. 11171/ENV/09-747/620/09-ZS 25.

We performed full-body necropsies, which included examination of the subcutaneous tissue, body cavity, esophagus, stomach, intestines, cloaca, bursa of Fabricius, liver, gall bladder, spleen, lungs, trachea, bronchus, air sacs, kidneys, and oviducts using a stereomicroscope. We fixed helminths in 70% ethanol, stained them with borax carmine, transferred them through an alcohol series to xylene, and mounted them in Canada balsam as described previously [27]. We identified the stained specimens using taxonomic keys [28–32], also reflecting recent reclassifications. We recorded the abundance and species richness of trematodes in each examined host individual [33]. We stored representative specimens in the Comenius Museum collections (Přerov, Czech Republic). Most of the new host–parasite records from the examined data sets were published in our previous studies, and some of the analyzed helminths were already used for molecular analyses [34–39]. The nomenclature follows the Fauna Europaea database [40] and recently published reclassifications [34, 35, 39, 41]. For details concerning the life cycles of the examined parasites, refer to Sitko et al. [42].

We calculated basic characteristics of the analyzed component communities (mean frequency of infection and helminth load) and trematode species-specific mean relative prevalence and mean intensity of infection. We referred to the most prevalent trematodes in each host as dominant. We computed rarefaction curves based on the log gamma function for computing combinatorial terms to interpolate the trematode species richness data [43]. To extrapolate the trematode species richness, and therefore to estimate the true trematode species richness of the analyzed population, we calculated the Chao-1 estimator corrected for unseen trematode species [44, 45]. We further calculated the following variables: (1) the total number of trematode species found, (2) the total number of individuals found, (3) the trematode species prevalence (the proportion of host individuals infected by trematodes), and (4) the intensity of infection (the number of trematode individuals per host calculated over all individuals that were positive for the respective trematodes). We compared the trematode species richness using the Sørensen similarity index (presence/absence-based index that assigns a greater weight to shared species than to those found in only one data set) and assessed the differences in trematode diversity between the study periods using the Shannon diversity *t*-test (compares Shannon *H* indices with a bias correction term proposed by Poole [46] of two abundance data sets assuming equal sampling conditions). We compared the prevalence of dominant trematodes in the ≤ 2000 and 2011–2020 data sets by *χ*^2^ tests, and the intensity of infection of the ≤ 2000 and 2011–2020 data sets by the Mann–Whitney rank-sum test (Shapiro–Wilk normality tests failed in all cases). In the Results section, we describe the trematode communities in more detail with regard to the host age, host sex, and year examined. However, as the resulting data were highly variable, the study was not sufficiently powered to test for possible differences at this scale. We performed all the calculations in SigmaPlot 12.0, EstimateS 9.1.0, and PAST 2.14. Data are shown as mean ± SD unless stated otherwise; data for the intensity of infection are shown as mean ± SE.

## Results

### Total numbers of trematodes

We collected a total of 15,549 individuals belonging to 33 species of trematodes, which represented host-species-specific component communities in *C. ciconia* (9751 individuals, 9 trematode species; Additional file 1: Table S7), *P. pica* (239 individuals, 8 trematode species; Additional file 1: Table S8), *A. otus* (948 individuals, 4 trematode species; Additional file 1: Table S9), *B. buteo* (4363 individuals, 10 trematode species; Additional file 1: Table S10), *F. tinnunculus* (69 individuals, 5 trematode species; Additional file 1: Table S11), and *V. vanellus* (179 individuals, 8 trematode species; Additional file 1: Table S12).

### Changes at the level of individual host species

**Fig. 1:**
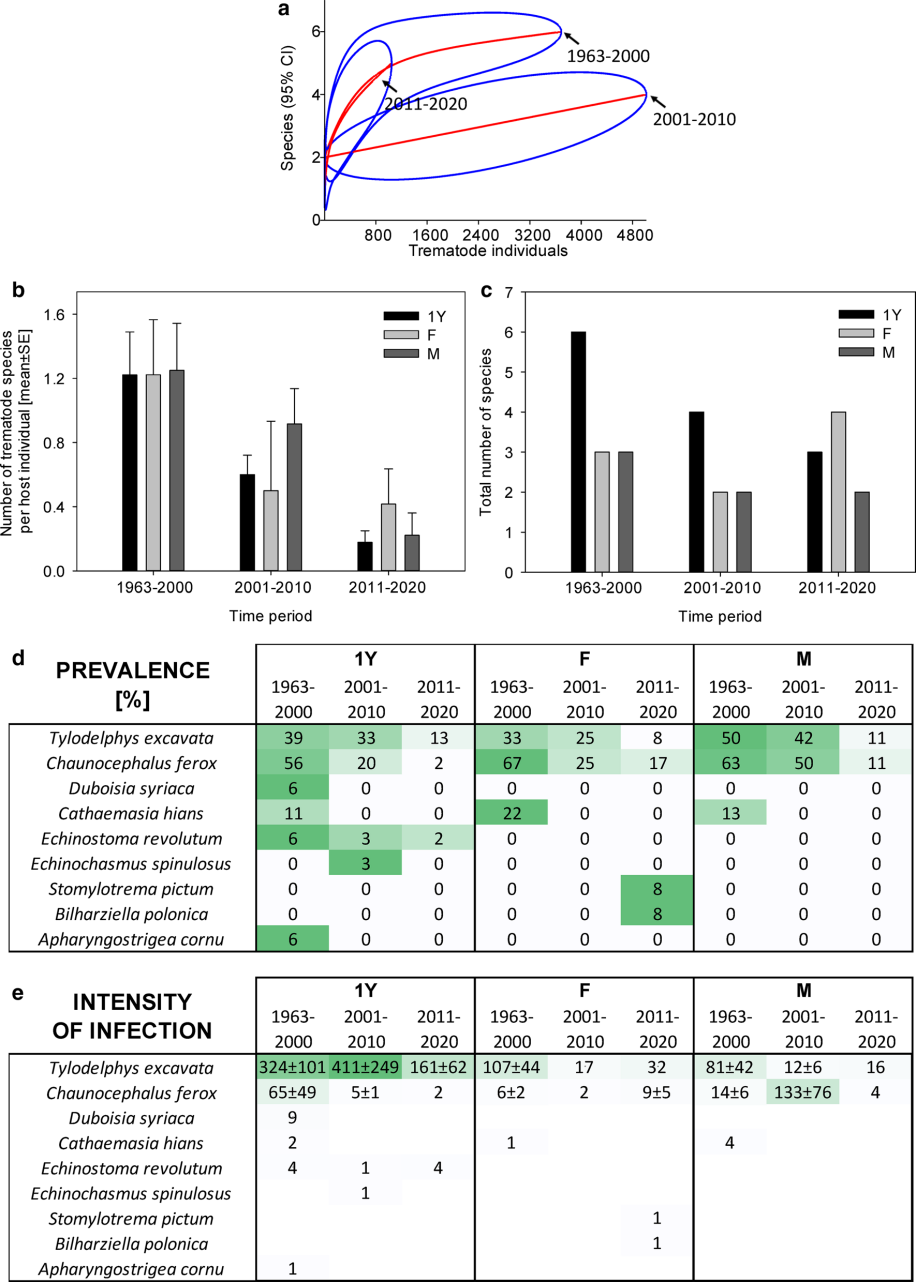
Dynamics of trematode assemblages associated with the Czech population of *Ciconia ciconia*. (**a**) The rarefaction curves (red; 95% confidence intervals in blue) of component communities in *C. ciconia* calculated separately for each of the four study periods (1963–2000, 2001–2010, 2011–2020). Dynamics of changes in the number of trematode species per host individual (**b**), the total number of trematode species found (**c**), and the prevalence (**d**) and the intensity of infection ± SE (**e**). The data for the prevalence and the intensity of infection are shown as a heatmap; the prevalence is shown as a percent of infected hosts, with the color green assigned to the highest prevalence of the respective trematode species and white assigned to zero prevalence. A similar color code of the heatmap was used to visualize the intensity of infection; however, the whole color code scale of the intensity of infection is based on all fields within the heatmap. Source data are provided in Additional file 1: Table S7

**Fig. 2:**
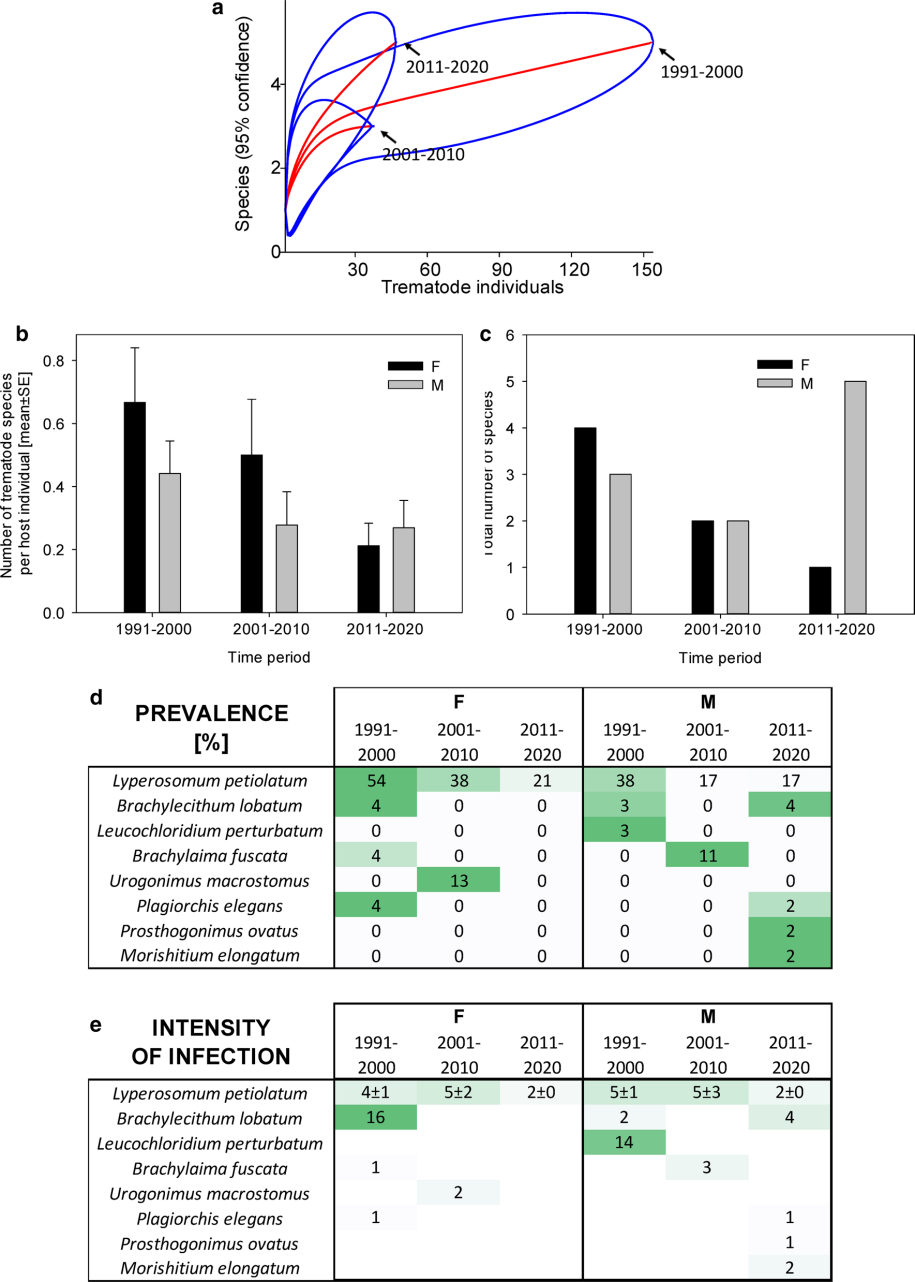
Dynamics of trematode assemblages associated with the Czech extravilan population of *Pica pica*. (**a**) The rarefaction curves (red; 95% confidence intervals in blue) of component communities in *P. pica* calculated separately for each of the three study periods (1991–2000, 2001–2010, 2011–2020). Dynamics of changes in the number of trematode species per host individual (**b**), the total number of trematode species found (**c**), and the prevalence (**d**) and the intensity of infection ± SE (**e**). The data for the prevalence and the intensity of infection are shown as a heatmap; the prevalence is shown as a percent of infected hosts, with the color green assigned to the highest prevalence of the respective trematode species and white assigned to zero prevalence. A similar color code of the heatmap was used to visualize the intensity of infection; however, the whole color code scale of the intensity of infection is based on all fields within the heatmap. Source data are provided in Additional file 1: Table S8

**Fig. 3:**
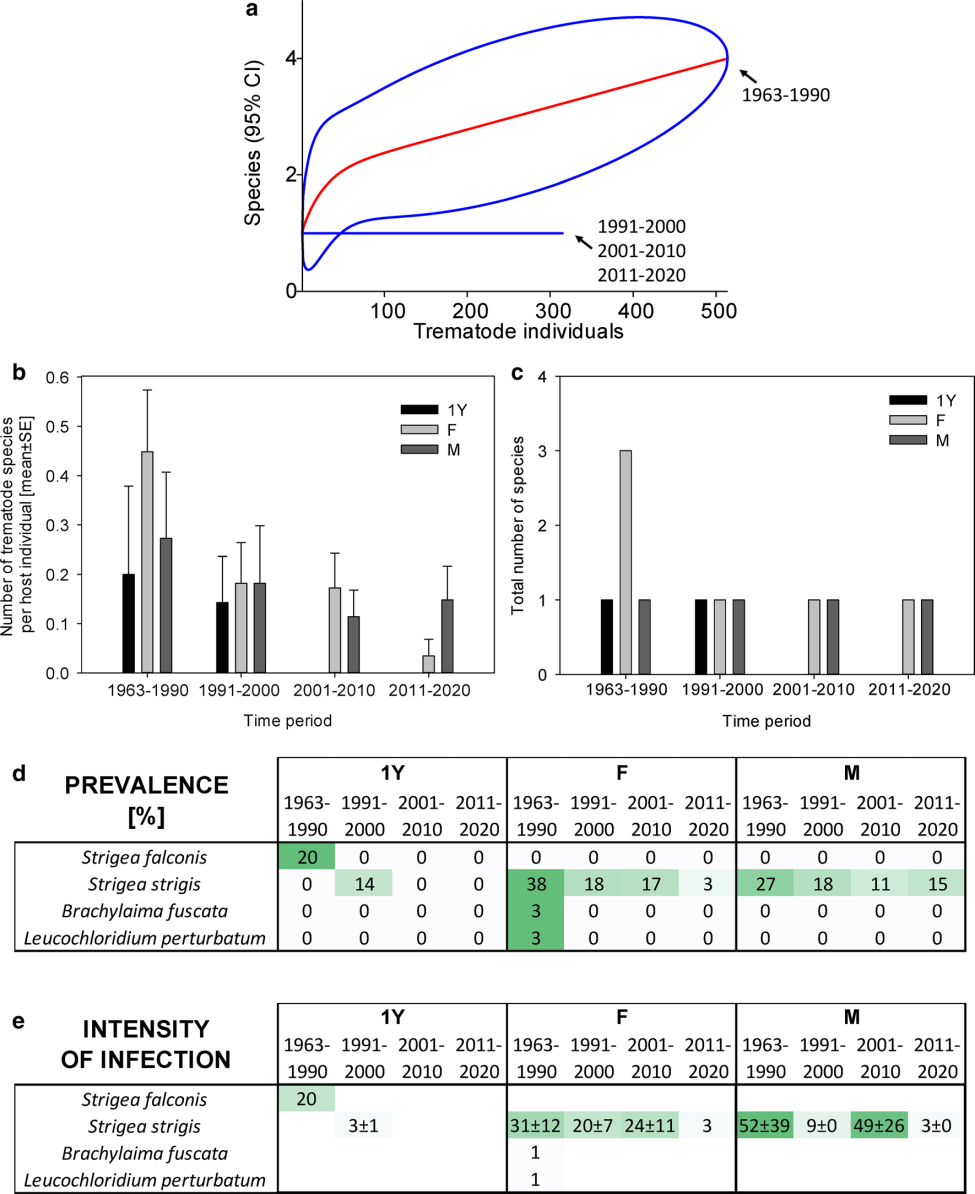
Dynamics of trematode assemblages associated with the Czech population of *Asio otus*. (**a**) The rarefaction curves (red; 95% confidence intervals in blue) of component communities in *A. otus* calculated separately for each of the four study periods (1963–1990, 1991–2000, 2001–2010, 2011–2020). Dynamics of changes in the number of trematode species per host individual (**b**), the total number of trematode species found (**c**), and the prevalence (**d**) and the intensity of infection ± SE (**e**). The data for the prevalence and the intensity of infection are shown as a heatmap; the prevalence is shown as a percent of infected hosts, with the color green assigned to the highest prevalence of the respective trematode species and white assigned to zero prevalence. A similar color code of the heatmap was used to visualize the intensity of infection; however, the whole color code scale of the intensity of infection is based on all fields within the heatmap. Source data are provided in Additional file 1: Table S9

**Fig. 4:**
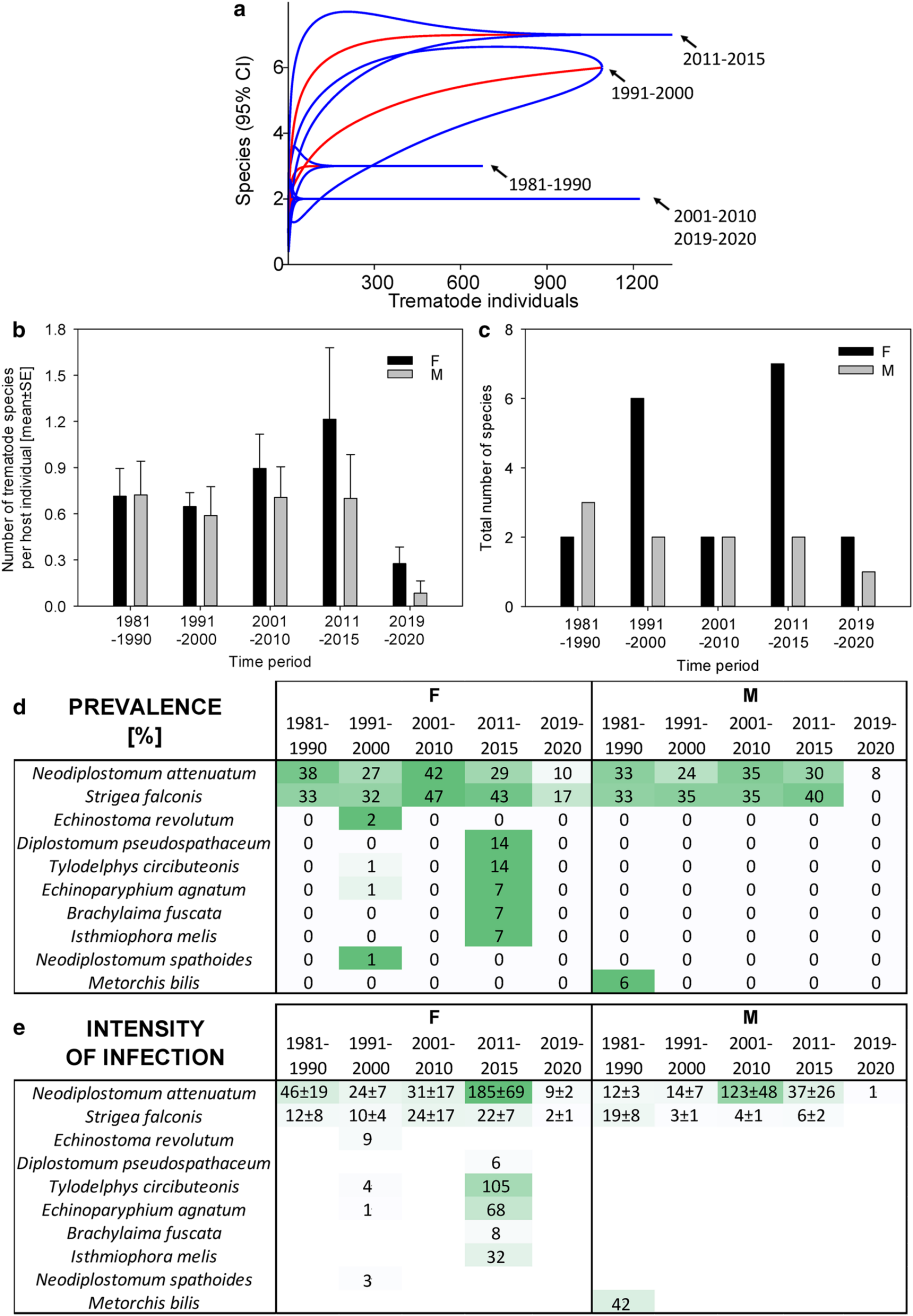
Dynamics of trematode assemblages associated with the Czech winter population of *Buteo buteo*. (**a**) The rarefaction curves (red; 95% confidence intervals in blue) of component communities in *B. buteo* calculated separately for each of the five study periods (1981–1990, 1991–2000, 2001–2010, 2011–2020). Dynamics of changes in the number of trematode species per host individual (**b**), the total number of trematode species found (**c**), and the prevalence (**d**) and the intensity of infection ± SE (**e**). The data for the prevalence and the intensity of infection are shown as a heatmap; the prevalence is shown as a percent of infected hosts, with the color green assigned to the highest prevalence of the respective trematode species and white assigned to zero prevalence. A similar color code of the heatmap was used to visualize the intensity of infection; however, the whole color code scale of the intensity of infection is based on all fields within the heatmap. Source data are provided in Additional file 1: Table S10

**Fig. 5:**
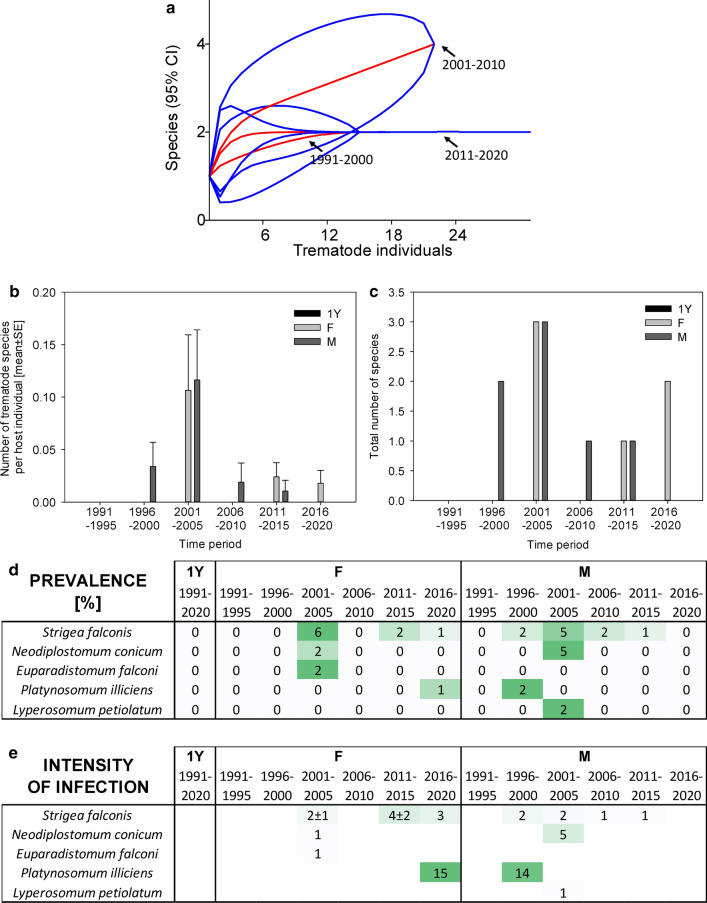
Dynamics of trematode assemblages associated with the Czech population of *Falco tinnunculus*. (**a**) The rarefaction curves (red; 95% confidence intervals in blue) of component communities in *F. tinnunculus* calculated based on birds that were examined in study periods 1991–2000, 2001–2010, and 2011–2020. Dynamics of changes in the number of trematode species per host individual (**b**), the total number of trematode species found (**c**), and the prevalence (**d**) and the intensity of infection ± SE (**e**). The data for the prevalence and the intensity of infection are shown as a heatmap; the prevalence is shown as a percent of infected hosts, with the color green assigned to the highest prevalence of the respective trematode species and white assigned to zero prevalence. A similar color code of the heatmap was used to visualize the intensity of infection; however, the whole color code scale of the intensity of infection is based on all fields within the heatmap. Source data are provided in Additional file 1: Table S11

**Fig. 6:**
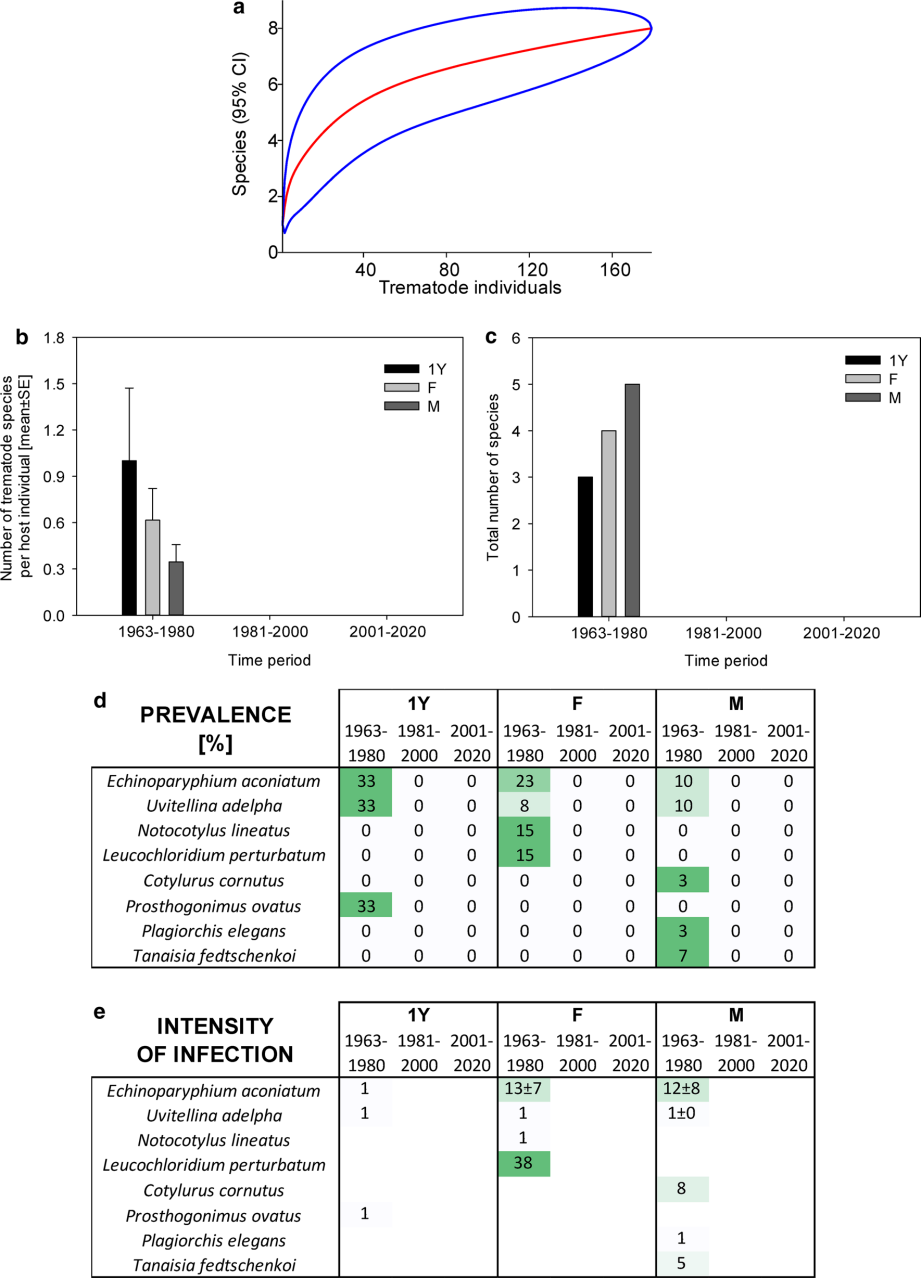
Dynamics of trematode assemblages associated with the Czech population of *Vanellus vanellus*. (**a**) The rarefaction curves (red line; 95% confidence intervals in blue) of component communities in *V. vanellus* calculated only for the first sampling period (1963–1980), as there were no species acquired in later time periods (1981–2000, 2001–2020). Dynamics of changes in the number of trematode species per host individual (**b**), the total number of trematode species found (**c**), and the prevalence (**d**) and the intensity of infection ± SE (**e**). The data for the prevalence and the intensity of infection are shown as a heatmap; the prevalence is shown as a percent of infected hosts, with the color green assigned to the highest prevalence of the respective trematode species and white assigned to zero prevalence. A similar color code of the heatmap was used to visualize the intensity of infection; however, the entire color code scale of the intensity of infection is based on all fields within the heatmap. Source data are provided in Additional file 1: Table S12

Trematodes in the examined host species displayed strong differences in population trends. We observed dramatic population losses of all trematode species in *C. ciconia* (Fig. 1), in which the number of trematode species per host individual declining from 1.2 ± 1.0 in 1963–2000 to 0.7 ± 0.7 in 2001–2010 and 0.2 ± 0.5 in 2011–2020. We observed less extensive declines in helminths in *P. pica* (Fig. 2) and *A. otus* (Fig. 3). In *B. buteo*, the only two characteristic trematode species were present until the period 2011–2015, declining to a third or less of their original prevalence only in recent years (Fig. 4). In this species, the number of trematode species per host individual declined from 1.0 ± 1.5 in 2011–2015 to 0.2 ± 0.5 in 2019–2020. In *F. tinnunculus*, the prevalence of trematodes was negligible from the very beginning of the study period (Fig. 5). In *V. vanellus*, we found trematodes only in host individuals examined prior to 1980, when the number of trematode species per host individual was at 0.5 ± 0.7. We did not find any trematodes in *V. vanellus* after that time, despite examining 33 host individuals of various sex and age during the period 1981–2020. Rarefaction analyses indicated that the trematode assemblages of five of the six birds consisted of lower numbers of trematode species when compared to those analyzed in the same study area one or more decades earlier (Figs. 1, 2, 3, 4, and 6); only the data from *F. tinnunculus* indicated no decline; however, the vast majority of examined *F. tinnunculus* were free of any trematodes already at the beginning of the study period (Fig. 5).

**Fig. 7:**
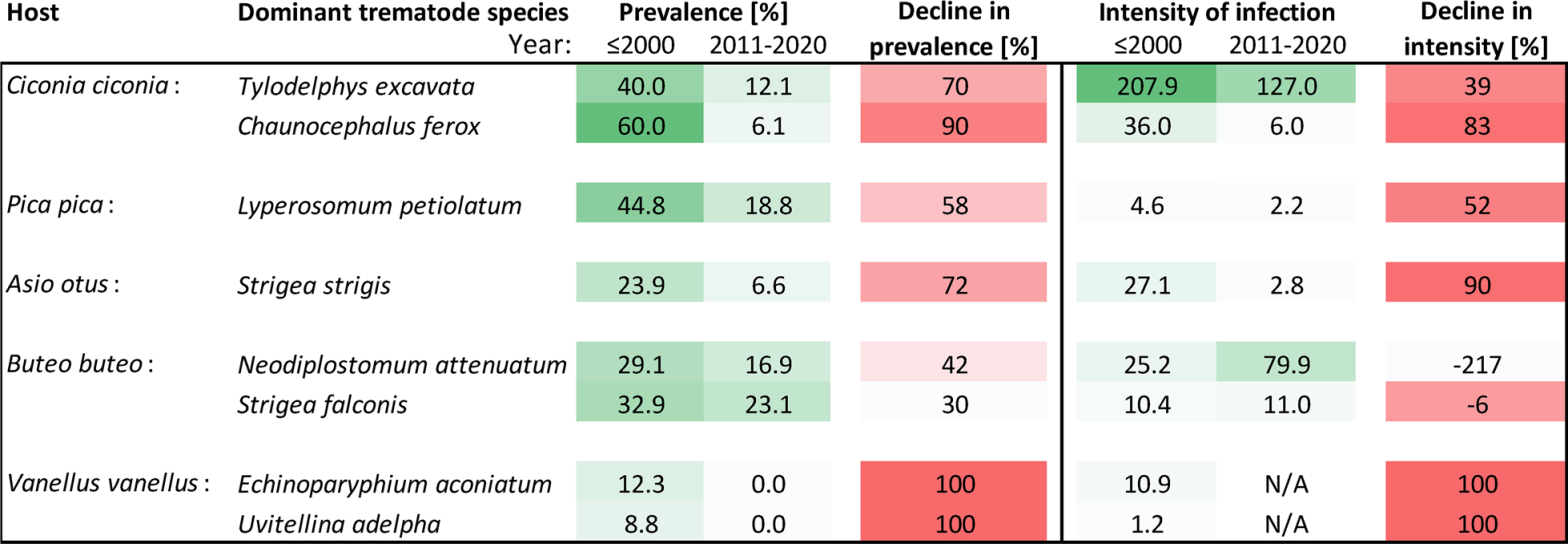
Overview of changes in the prevalence of previously dominant trematode species found in 2000 or earlier as compared to the period 2011–2020. The prevalence in the two respective time periods and the decline in prevalence of the respective dominant species are indicated. The prevalence is shown as a percent of infected hosts, with the color green assigned to the highest prevalence of the respective trematode species and white assigned to zero prevalence. All the observed declines in prevalence were found to be significant (*χ*^2^ test *P* < 0.001 each); the declines in trematodes of *V. vanellus* were not tested. Using the same logic, the figure shows the intensity of infection by the previously dominant trematode species in the two respective time periods and the change in the intensity of infection of the respective dominant species. The color code used follows similar logic as that for the prevalence. All the observed changes in the intensity of infection were tested by the Mann–Whitney rank-sum test, which revealed that only the differences in the intensity of infection by *Lyperosomum petiolatum* and *Strigea strigis* were significant (*P* < 0.05 each); the changes in the intensity of infection in *V. vanellus* were not tested

Despite a sharp decrease in helminth abundance, the Chao-1 trematode species richness in *C. ciconia*, *P. pica*, *B. buteo*, and *F. tinnunculus* did not decrease (Table 2). In contrast, three of the four species were present in *A. otus* only prior to 1990, which translated into a sharp decline of the Chao-1 trematode species richness from 5.0 ± 2.2 in 1963–1990 to 1.0 ± 0.0 trematode species in all subsequent study periods. In *V. vanellus*, the Chao-1 trematode species richness reached 8.5 ± 1.3 in 1963–1980. After 1980, the examined *V. vanellus* did not contain any single trematode individual; therefore, the species richness for the later time periods cannot be estimated (Table 2). A comparison of diversity using the Sørensen similarity index and Shannon diversity *t*-test is provided in Table 2. The rarefaction analyses and the Chao-1 species richness estimator point to the fact that the overall trematode species richness of the study bird hosts changed only to a limited extent. However, this perceived stability of the study assemblages was can be explained by the isolated records of trematode species that were likely acquired from rarely exploited food sources, while the previously dominant species which were characteristic of the examined bird species declined (Fig. 7), as shown using the trematode-specific analyses below.

**Table 2:** Comparison of trematode species richness and diversity of the analyzed component communities in the 2010s and earlier time periods, using the Sørensen similarity index and Shannon diversity *t*-test

	*Ciconia ciconia*	*Pica pica*	*Asio otus*	*Buteo buteo*	*Falco tinnunculus*	*Vanellus vanellus*
Compared time periods	1963–2000 vs. 2011–2020	1991–2000 vs. 2011–2020	1963–1990 vs. 2011–2020	1981–1990 vs. 2019–2020	1991–2000 vs. 2011–2020	1963–1980 vs. 1981–2020
Shannon diversity *t*-test (*t*; *df*; *P*)	16.0; 1663.6; < 0.001	−0.4; 74.6; 0.72	5.9; 514.0; < 0.001	3.1; 43.5; 0.003	−2.0; 16.6; 0.07	N/A (Σ_*n*2_ = 0)
Sørensen similarity index	0.545	0.600	0.400	0.800	1.000	0.000
Number of trematode species found in both study periods	3	3	1	2	2	0
Number of trematode species found only in the first study period	3	2	3	1	0	8
Number of trematode species found only in the last study period	2	2	0	0	0	0
Number of host individuals examined (first/last study period)	35/66	58/85	45/76	39/41	211/515	16/33
Number of trematode individuals found (first/last study period)	3690/1046	154/47	514/14	678/37	16/31	179/0
Number of trematode species found (first/last study period)	6/5	5/5	4/1	3/2	2/2	8/0
Intensity of infection (first/last study period)	113.1/15.9	2.7/0.6	11.4/0.2	17.4/0.9	0.08/0.06	11.2/0.00
Chao-1 (first/last study period)	6.0 ± 0.5/6.0 ± 2.2	6.0 ± 2.2/5.5 ± 1.3	5.0 ± 2.2/1.0 ± 0.0	1.0 ± 0.0/2.0 ± 0.0	2.0 ± 0.3/2.0 ± 0.0	8.5 ± 1.3/N/A (0)

The numbers of trematode species that were found at both analyzed time points and those that were found only in one of the indicated time periods are shown

The decrease in the diversity of component communities from the earliest to the most recent available time periods was statistically significant in four of the six examined species (see Table 2 for more detail). The exceptions were the trematodes of *P. pica* and *F. tinnunculus* (Table 2). In *P. pica*, there was a decline in trematode species associated with the examined host in the first study period (1991–2000), but other trematode species were newly recorded in this species only in recent years. The second species in which there were no significant dynamics in the diversity of associated trematode communities, *F. tinnunculus*, generally had a low number and prevalence of trematodes, and we recorded both species associated with *F. tinnunculus* in roughly the same prevalence and intensity of infection across all the examined time periods (Fig. 5).

### Species-specific changes

In *C. ciconia*, two trematode species, *Tylodelphys excavata* and *Chaunocephalus ferox*, were characteristically considered dominant; the first species had 40.0% prevalence prior to 2000 and the latter species had 60.0% prevalence prior to 2000. However, their prevalence decreased in 2001–2010 and further declined in 2011–2020, when the prevalence reached 12.1% and 6.1%, representing a decline of 70% and 90% (*χ*^2^ test *P* < 0.001 each; Fig. 1). Despite the sharp declines in prevalence, the intensity of infection by *T. excavata* did not change, and reached 208 ± 61 individuals per host in the ≤ 2000 period and 127 ± 51 individuals per host in 2011–2020 (Mann–Whitney rank-sum test *P* > 0.05; *n*_1_ = 14, *n*_2_ = 8, *T* = 80, *U* = 44). There was a similar trend in *C. ferox*, the incidence of which reached 36 ± 24 individuals per host in the ≤ 2000 period and 6 ± 3 individuals per host in 2011–2020 (Mann–Whitney rank-sum test *P* > 0.05; *n*_1_ = 21, *n*_2_ = 4, *T* = 37.5, *U* = 27.5). Another characteristic parasite species of storks, *Cathaemasia hians*, was completely absent in the examined *C. ciconia* after 2001. Isolated records of two species, *Stomylotrema pictum* and *Bilharziella polonica*, each represented by a single individual, were the only species that were recorded for the first time in the most recent study period 2011–2020 (Fig. 1).

In *P. pica*, the dominant trematode species, *Lyperosomum petiolatum*, was characteristically found in 44.8% of examined host individuals before 2000. However, its prevalence declined in 2011–2020 by 58% to only 18.8% (*χ*^2^ test *P* < 0.001; Fig. 2). The intensity of infection by *L. petiolatum* decreased from 4.6 ± 0.6 individuals per host in the ≤ 2000 period to 2.2 ± 0.3 individuals per host in 2011–2020 (Mann–Whitney rank-sum test *P* = 0.007; *n*_1_ = 26, *n*_2_ = 16, *T* = 240.5, *U* = 140.5). Other trematode species were present in low prevalence, with no evidence for their decline. Isolated records of two species, *Prosthogonimus ovatus* and *Morishitium elongatum*, each found in a single host individual, were the only species that were recorded for the first time in the most recent study period 2011–2020 (Fig. 2).

In *A. otus*, we found only four trematode species. The dominant trematode species of adult *A. otus*, *Strigea strigis*, was characteristically found in about a third of examined host individuals before 1990. However, its prevalence gradually declined from 23.9% to just 6.6% (*χ*^2^ test *P* < 0.001; Fig. 3). The intensity of infection by *S. strigis* declined correspondingly to just one tenth of the original intensity of infection, from 27.1 ± 8.7 individuals per host in the ≤ 2000 period to 2.8 ± 0.3 individuals per host in 2011–2020 (Mann–Whitney rank-sum test *p* = 0.036; *n*_1_ = 22, *n*_2_ = 5, *T* = 36, *U* = 21). Three other three trematode species were present in low prevalence; we found all of them in the first study period before 1990. Other than *S. strigis*, we did not find any trematode species in *A. otus* that were examined in 1991–2020 (Fig. 3).

In *B. buteo*, there were two trematode species with high prevalence. These were *Neodiplostomum attenuatum* and *Strigea falconis*, which were present in 29.1% and 32.9% of examined *B. buteo* prior to 2000. Their prevalence remained similar until 2011–2015; the intensity of infection was generally high, showing strong fluctuations. However, the two trematode species were associated with only a fraction of the original numbers of *B. buteo* in its most recently examined cohort from 2019–2020, with the intensity of infection also declining to the lowest values (Fig. 4). The differences in *N. attenuatum* and *S. falconis* prevalence between the ≤ 2000 cohort and 2011–2020 cohort were statistically significant (*χ*^2^ test *P* < 0.001 each). There were no significant changes in the incidence of these two species. In *N. attenuatum*, the incidence reached 25 ± 6 individuals per host in the ≤ 2000 period and 80 ± 36 individuals per host in 2011–2020 (Mann–Whitney rank-sum test *P* > 0.05; *n*_1_ = 46, *n*_2_ = 11, *T* = 356.5, *U* = 215.5). In *S. falconis*, the incidence reached 10 ± 3 individuals per host in the ≤ 2000 period and 11 ± 4 individuals per host in 2011–2020 (Mann–Whitney rank-sum test *P* > 0.05; *n*_1_ = 52, *n*_2_ = 15, *T* = 517, *U* = 383). We found eight other trematode species in *B. buteo*; however, these were all rather isolated records, although sometimes at high intensity of infection, such as in the case of *Tylodelphys circibuteonis* and *Echinoparyphium agnatum* (Fig. 1).

First-year birds of *F. tinnunculus* were free of any trematodes; adult *F. tinnunculus* were rarely infected by five species of trematodes, which did not display any seasonal trends. We found the highest prevalence of infections in 2001–2005; there were no species that would newly emerge in the study periods after 2006 (Fig. 5).

In *V. vanellus*, we recorded eight species of trematodes in the first study period prior to 1980. The *V. vanellus* trematodes were dominated by *Echinoparyphium aconiatum* (prevalence 12.3%, intensity of infection 11 ± 5 individuals per host) and *Uvitellina adelpha* (prevalence 8.8%, intensity of infection 1.2 ± 0.2 individuals per host). After 1980, all examined *V. vanellus* were free of any trematodes (Fig. 6).

## Discussion

The present study provides another important piece of evidence of the simplification of helminth communities. The data complement our previous study on wetland birds [12] by showing that trematode species that have dominated the component communities in various birds that use farmlands as their feeding habitats have experienced massive declines, and in some cases they have completely vanished from the analyzed bird host populations. These changes have affected in particular the previously dominant trematode species that had been characteristic of the examined host bird species (Fig. 7).

We assumed that the population changes in common farmland birds could be associated with changes in the assemblages of trematodes, as their presence is directly linked to the preservation of long-established food webs and habitat use adaptations of their hosts [47–49]. The most prominent changes were, indeed, associated with the trematodes of host species, which experienced a strong population decline (*V. vanellus*). However, strong effects were also found in bird species, which recently shifted their feeding strategies and correspondingly shifted the spectrum of captured prey (*C. ciconia*) [17, 18]. In contrast, the species richness and diversity of trematodes of some other species (*F. tinnunculus*) changed very little throughout the study period, and displayed only fluctuations. These fluctuations may reflect generally low prevalence of the *F. tinnunculus* trematodes, which means that very large cohorts of *F. tinnunculus* would need to be examined to provide a definitive answer on whether these fluctuations were artifacts, or whether they reflected real changes in the *F. tinnunculus* trematode assemblages (Fig. 5). The association between changes in trematode prevalence and changes in feeding strategies of their host birds points to the fact that a large part of observed changes could likely be determined by changes in the composition of intermediate host assemblages and prey preferences of the birds [50–52]. However, the declines in trematodes were widespread, involved multiple bird orders, and involved both common and rare trematode species. In particular, the changes affected the previously dominant trematode species of the birds that use farmlands as their feeding habitats. In contrast, with the exception of *V. vanellus*, the frequency and spectrum of isolated records of trematode species did not change to any large extent. This is likely related to the random nature of their acquisition from rarely exploited food sources, particularly from food sources associated with wetlands, as many of these trematodes use various wetland snails as their intermediate hosts.

We recently reported similar or even stronger declines in trematodes in freshwater aquatic birds [12]. The changes identified in birds that use farmlands as their feeding habitats differed from those identified previously in freshwater aquatic birds, particularly in *Anas platyrhynchos*. The component communities of *A. platyrhynchos* faced simplification and dominance by only a few surviving species. The two other previously examined freshwater aquatic birds, *Fulica atra* and *Chroicocephalus ridibundus*, displayed a mixed response, with severe declines in previously dominant species and overall simplification of the trematode component communities [12].

All the available data are only observational; therefore, the causes of observed declines are unclear. In *C. ciconia*, the changes could be related to a decline in the contribution of wetland organisms to the diet of this host species. The increasing contribution of anthropogenic sources of diet also could play a role [53, 54]. Besides the changes in feeding habits, the decline in trematode prevalence and intensity of infection could be related to the changes in farm management and land use since the 1950s, including agricultural intensification and landscape simplification [55]. The common agricultural policy of the European Union and the collectivism practiced in the eastern part of Europe further accelerated the loss of uncultivated elements, such as hedgerows, woodlots, and ditches [56], resulting in a decline in farmland landscape biodiversity [57–59].

An important but under-researched factor is the broad use of azole fungicides in agriculture, which has shaped communities of various non-target organisms [60–62]. Some of the azole fungicides, such as carbendazim, are among the most frequently used agrochemicals worldwide, despite having already been banned in the USA and the European Union [63]. The use of azole fungicides is directly linked to the abundance of helminths, as the same compounds belong to the most effective antiparasitic treatments. In veterinary medicine, albendazole or mebendazole are commonly used for this purpose [64–67]; however, many other azole compounds display antihelminthic activity of similar extent due to their shared mechanism of action that is based on the binding to β-tubulin in microtubules [68].

The diversity and abundance of parasites generally decreases with human-induced habitat disturbance [69–73]. The disruption of spatiotemporal relationships within host assemblages likely facilitated a decline in species richness of trematode assemblages. Surprisingly, we only rarely identified severe intensity of infection of the respective trematodes, and we found no outbreaks of trematode species. This corresponds to the fact that the loss of diversity was accompanied by a decline in the intensity of infection. The decline in the intensity of infection affected the trematode assemblages of *V. vanellus*, *A. otus*, and *B. buteo* most severely; in all three species, the intensity of infection declined by more than one order of magnitude. Infection intensity also declined several times in *C. ciconia* and *P. pica*, and maintained the same level only in *F. tinnunculus*, where it was very low throughout the study period. The trematode biodiversity loss was rather gradual, and included the decline in both the prevalence and the intensity of infection in the respective trematode species. The diversity of the trematode assemblages differed strongly among four of the six bird host species examined when comparing the assemblages in 1963–2000 with those examined in 2011–2020; the exceptions were *P. pica* and *F. tinnunculus*. In general, we observed a simplification of the trematode assemblages; in three of the six analyzed host species, some or all the trematode species were present prior to 2000 but not in the 2011–2020 period. However, there was no such change in *F. tinnunculus*, and both lost and newly acquired species were present in *C. ciconia* and *P. pica* (Table 2).

## Limitations

A limitation of the present study is that it relied on an opportunistic sampling design, where the carcasses of five of the six examined species consisted solely of wounded or injured individuals, and therefore may not necessarily represent the helminths that would be present in birds of good health. Further, we cannot exclude the possibility that trematode species with low prevalence might escape detection in any of the study periods due to limited number of examined host individuals. However, the purpose of the study was to illustrate the overall longitudinal trends in trematode component communities, not to provide a complete list of species that infected the examined host species. Although the present study is based on one of the largest data sets of host individuals among the studies of bird trematodes, it still suffers from issues associated with limited sample size. The sampling effort affects not only trematode richness, but also prevalence, intensity, and some of the diversity indices. Especially at low prevalence, intensity estimates can be skewed when sample size is small. We also cannot exclude the possibility that the observed population trends may differ from those that would be experienced in other regions with better-preserved farmland habitats. As we have shown in Additional file 1: Tables S1–S6 [19–24, 36, 37, 74–107], there are strong differences in the prevalence of trematodes among different regions within host distribution ranges. However, the present study, which was based on definitive hosts, confirmed the declines in trematodes reported in previous longitudinal studies on the diversity of trematodes in intermediate hosts in other regions [6–10].

## Conclusions

The analysis of six unrelated common bird species that use farmlands as their feeding habitats revealed a previously unreported collapse of formerly dominant trematode species. These trematode species declined in terms of both prevalence and intensity of infection. In contrast, isolated records of a broad range of other trematode species continued to be detected throughout the study period, which is likely related to occasional use of a broad spectrum of food sources. However, the collapse of host–parasite networks in common bird species that use farmlands as their feeding habitats points to yet unknown factors that underlie such dramatic changes. As we observed most of the declines in recent decades, we speculate that the use of recently developed agrochemicals, perhaps the azole fungicides, also known as anthelminthic agents, may be responsible for the unprecedented decline in the farmland trematodes.

## Supplementary Information

**Additional file 1: Table S1.** Previous records of trematodes from *Ciconia ciconia*. **Table S2.** Previous records of trematodes from *Pica pica.* **Table S3.** Previous records of trematodes from *Asio otus*. **Table S4.** Previous records of trematodes from *Buteo buteo*. **Table S5.** Previous records of trematodes from *Falco tinnunculus*. **Table S6.** Previous records of trematodes from *Vanellus vanellus*. **Table S7.** Raw data. Listed are all examined *C. ciconia* host individuals together with the abundance of all trematode species found. Indicated are the age and sex of analyzed hosts, as well as the year when the respective host individual was acquired. **Table S8.** Raw data. Listed are all examined *P. pica* host individuals together with the abundance of all trematode species found. Indicated are the age and sex of analyzed hosts, as well as the year when the respective host individual was acquired. **Table S9.** Raw data. Listed are all examined *A. otus* host individuals together with the abundance of all trematode species found. Indicated are the age and sex of analyzed hosts, as well as the year when the respective host individual was acquired. **Table S10.** Raw data. Listed are all examined *B. buteo* host individuals together with the abundance of all trematode species found. Indicated are the age and sex of analyzed hosts, as well as the year when the respective host individual was acquired. **Table S11.** Raw data. Listed are all examined *F. tinnunculus* host individuals together with the abundance of all trematode species found. Indicated are the age and sex of analyzed hosts, as well as the year when the respective host individual was acquired. **Table S12.** Raw data. Listed are all examined *V. vanellus* host individuals together with the abundance of all trematode species found. Indicated are the age and sex of analyzed hosts, as well as the year when the respective host individual was acquired.

## Data Availability

Representative specimens of the helminths analyzed in this study are available in the collections of the Comenius Museum in Přerov. All data are available in the main text or additional materials.

## Abbreviations

1Y: First-year birds
IUCN: International Union for Conservation of Nature
